# Evaluation of the Anti-Müllerian Hormone and its Association with Embryo Quality in Advanced Reproductive Treatments in a Latin American Population

**DOI:** 10.5935/1518-0557.20210050

**Published:** 2022

**Authors:** Héctor Salvador Godoy Morales, Germán Gabriel Palacios López, Daniel Vieyra Cortés, Griselda Claribel Reyes Torres, Hilda Sanchez Hernández, Miguel Loyo Guiot, Francisco Miguel Rojas Camacho, Gabriela Ayala Montoya, Berenice Flores Maldonado

**Affiliations:** 1 Head of Assisted Research and Treatment in Human Reproduction (ART) Reproductive Medicine Unit at Ángeles del Pedregal Hospital, Mexico City, Mexico; 2 Specialist in Human Reproductive Medicine and Master in Health Sciences, Mexico City, Mexico; 3 Specialist in Human Reproductive Medicine, Mexico City, Mexico; 4 Resident of Human Reproductive Medicine at Ángeles del Pedregal Hospital, Mexico City, Mexico

**Keywords:** anti-Müllerian hormone, ovarian reserve, embryo quality, advanced reproductive treatments

## Abstract

**Objective:**

Serum anti-Müllerian hormone (AMH) presents a strong positive correlation with quantitative aspects of the ovarian reserve, while its correlation with embryo quality is unclear. This study assessed the association between serum AMH as a marker of ovarian reserve and embryo quality, in women undergoing in vitro fertilization.

**Methods:**

This observational analytical retrospective study included patients seen between 2010 and 2018. In vitro fertilization patients with measured AMH levels were analyzed based on the following parameters: number of retrieved oocytes; number of metaphase II oocytes; embryo quality; and treatment outcome. Statistical analysis was performed using ANOVA, Mann-Whitney U test, linear regression, and Pearson and Spearman correlations.

**Results:**

We found a positive correlation between AMH levels, number of retrieved oocytes and number of metaphase II oocytes (r 0.649, *p*=0.000). The numbers of retrieved and metaphase II oocytes were predicted in 42% (R2: 429) of the cases based on AMH levels (*p*=0.000). Serum AMH levels were not associated with embryo quality on Day 3 (*p*=0.151); an association was seen between AMH levels and embryo quality on Day 5 (*p*=0.006). The distribution of AMH levels was the same across patients, regardless of whether they were able to achieve pregnancy (*p*=0.767).

**Conclusions:**

AMH levels correlated with embryo quality on Day 5; no association was found between AMH levels and embryo quality on Day 3 or pregnancy rate. The use of AMH levels to predict embryo quality still requires further studies; therefore, AMH should be used to assess the ovarian reserve only.

## INTRODUCTION

Anti-Müllerian hormone (AMH) is expressed after birth in the granulosa cells of healthy small growing follicles. It plays diverse roles in various stages of folliculogenesis, from the primordial follicle to the stages where follicles respond according to follicle stimulating hormone (FSH) levels (Weenen *et al*., 2004), and is also an indicator of ovarian reserve. It is an excellent predictor of response to controlled ovarian stimulation in in vitro fertilization (IVF)/intracytoplasmic sperm injection (ICSI) treatments (Godoy-Morales *et al*., 2012). Although a strong positive correlation between serum AMH levels and the number of oocytes retrieved during IVF has been reported (Gruijters *et al*., 2003; Godoy-Morales *et al*., 2012; Seifer *et al*., 2002), previous studies have not shown a clear correlation between AMH and embryo quality.

Since oocyte quantity and quality affect embryo quality and seem to be associated with aging (Doroftei *et al*., 2015), it has been postulated that AMH might also be correlated with embryo quality. Accordingly, AMH might predict the response of patients receiving IVF treatment to ovarian stimulation, as well as their chance of conceiving (Tremellen *et al*., 2005).

This study aimed to assess the association between ovarian reserve and embryo quality in Days 3 and 5 by examining the correlation between serum AMH levels as a marker of ovarian reserve and the ASEBIR (Association for the Study of Reproductive Biology) criteria as an indicator of embryo quality (Hurtado de Mendoza, 2015).

## MATERIAL AND METHODS

This observational analytical cross-sectional retrospective study included the records of patients treated at the "Assisted Research and Treatment in Human Reproduction (ART) Reproductive Medicine Unit" of the Ángeles del Pedregal Hospital in Mexico City between 2010 and 2018. Patients diagnosed with infertility of any type and for any factor, with no age limit, who had cycles with their own eggs and who had complete medical records were included.

The following were excluded: cases with insufficient information or that had not undergone high complexity reproductive treatments, and patients undergoing cycles with donor eggs or embryos. The type of protocol or medications used during controlled ovarian stimulation was not taken into account in the analysis of variables.

The variables analyzed were age, baseline hormone levels (AMH, FSH, estradiol), number of retrieved oocytes, number of mature oocytes (metaphase II), fertilized oocytes, fertilization technique, type of transfer (fresh and devitrification), days of stimulation, pregnancy rate, and live birth rate. The ASEBIR criteria were used to assess embryo quality. Data on hormone levels comprised tests performed on Days 2 or 3 of the cycle, all of which were carried out at the same LabCorp reference laboratory (CENAREM S.A. de C.V.).

The data presented in this study were collected from patient records. The sampling was of the conventional non-probabilistic type and the research method used was observation and data collection by means of a registry, using the instrument from the information collection card. For the statistical analysis of the data, software packages (Microsoft Excel^©^ and IBM SPSS Statistics^©^, Version 20) were used alongside descriptive and inferential statistics, using measures of central tendency and dispersion for quantitative variables and frequencies and proportions for qualitative variables.

Pearson's correlation coefficient and Spearman's rank correlation coefficient were used as non-parametric alternatives in the calculation of the correlations between quantitative variables following a normal distribution. Comparisons between mean values of independent variables were carried out using Student's T test and one-way ANOVA for quantitative variables following a normal distribution or the Mann-Whitney U or Kruskal Wallis test (depending on the number of groups) for variables not following a normal distribution. And finally, to predict the factors associated with the success of highly complex treatments, linear regression was used to calculate the coefficient of determination.

This study was authorized by the Research Committee of Ángeles del Pedregal Hospital and adhered strictly to the current guidelines of the General Health Law and Article 17 of Chapter I of the Ethical Aspects of Research in Human Beings. The authors had no conflict of interest to declare.

## RESULTS

A total of 231 patient records from the ART unit of Ángeles del Pedregal Hospital were analyzed. The patients were aged 36.1 (5.34) years on average. Since every couple is tested for AMH levels (including cases of male infertility factor), the mean age was relatively low. The most frequent type of infertility was primary (58%), the main reason for female infertility was advanced maternal age (>40 years), and patients had been infertile for 41.64 (35.16) months on average.

The patients included in the study were prescribed the antagonist protocol. Most (60%) were given recombinant FSH (Gonal-F^®^, Merck Pharmaceuticals) alone for ovarian stimulation or recombinant-LH (Luveris^®^, Merck Pharmaceuticals) (30%), followed by human menopausal gonadotropin (Merional^®^, IBSA) (17.3%), with a follow-up visit at 5 days to assess dosage, which was defined based on conventional dose protocols. Once a follicle reached a diameter equal to or greater than 16 mm, the patient was started on the antagonist of choice. The drugs used were cetrorelix acetate (Cetrotide^®^, Merck Pharmaceuticals) in 93% of the cases and ganirelix acetate (Orgalutran^®^, MSD) in the remaining cases. In the case of the trigger to induce ovulation (which was indicated once a follicle reached 20 mm), the most frequently used drug was human chorionic gonadotropin (Choriomon^®^, IBSA) (45%), followed by recombinant hCG (Ovidrel^®^, Merck Pharmaceuticals) (35%), and gonadotropin-releasing hormone (GnRH) agonist (Gonapeptyl Daily^®^, Ferring) (hyper-responders or patients suspected for ovarian hyperstimulation syndrome) in doses of 0.2 mg (20%). Stimulation cycles lasted for 10.83 (±2.42) days on average.

An analysis was performed to assess whether the level of AMH correlates with embryo quality. Forty-six patients were excluded from the original sample (for not having embryos available for transfer) due to poor or lack of response to ovarian stimulation, and 185 were ultimately included in the study. The most frequent insemination technique to obtain embryos was ICSI (28.7%), followed by IVF (22.2%), IVF/ICSI (18.3%), intracytoplasmic morphologically selected sperm injection (IMSI) (6.5%), physiological intracytoplasmic sperm injection (PICSI) (3.9%), and IMSI/ICSI (.9%). We could not obtain data for 8% of the cases, and found a fertilization rate of 78%. Day 3 was the most frequent day of embryo transfer, with fresh embryo transfers accounting for 62.7% of the cases and frozen embryo transfers for 19%.

The analysis of the correlation between embryo quality and AMH levels revealed a negative weak relationship with obtaining quality A blastocysts (*p*=0.006) and no correlation with having good quality embryos on Day 3 (*p*=0.151), achieving pregnancy (*p*=0.747) or live births (*p*=0.842) (Table 1). When embryo quality parameters were analyzed for potential impacts on the ability to achieve pregnancy (regardless of AMH levels), we found that quality A embryos yielded higher pregnancy rates per cycle (52%), followed by quality B (33%), and quality C (31%) embryos, with a significant difference (*p*=0.002) between the three groups.

**Table 1.** Correlation between AMH variables and IVF cycle outcomes.

Parameter	Pearson or Spearman correlation (r)	*p* Value*
Retrieved oocytes	0.649	0.000
Metaphase II oocytes	0.648	0.000
Quality A embryos on Day 3	-0.106	0.151
Quality A blastocysts	-0.201	0.006
Pregnancy	0.024	0.747
Live newborn	0.013	0.842

* *p*<0.05 = statistically significant.

A third (33%) of the cycles analyzed in this study resulted in pregnancy and 22% in live births. AMH levels were similar between patients able and unable to achieve pregnancy (*p*=0.767).

## DISCUSSION

Differently from recent reports, the tests performed in this study showed that AMH was not associated with good quality embryos on Day 3, but with the number of good quality embryos on Day 5. Our findings are not in agreement with previous studies assessing the relationship between AMH and embryo quality using other methods, in which other authors did not find a relationship between AMH and embryo quality (Lie Fong *et al*., 2008).

An observational study by Smeenk *et al.* (2007) correlated baseline serum AMH levels with embryo quality in 112 women undergoing controlled ovarian stimulation and IVF/ICSI. Serum AMH failed to show a predictive capacity with respect to embryo quality. Lie Fong *et al*. (2008) reported on a correlation between baseline serum AMH and embryo quality in 125 women undergoing IVF. Women were randomly assigned to either mild stimulation or conventional stimulation and parameters were assessed in both groups. Although a positive correlation was seen between serum AMH and embryo quality in the mild stimulation group, no significant correlation was seen in the conventional stimulation group. The latter group of women is similar to the population enrolled in our study, and hence the results may be considered to be in agreement (Lie Fong *et al*., 2008).

Perhaps one of the most controversial findings in our study was that AMH levels correlated with embryo quality, as reported by De Conto *et al*. (2015); however, since our population was small, the quality A blastocyst higher pregnancy rate was not significantly different. It should also be mentioned that some authors have described AMH as a weak predictor for clinical pregnancy, which may explain why we found no differences between pregnancy rates in our results (Tal *et al*., 2015; Umarsingh *et al*., 2020). 

One of the limitations of this study is its retrospective nature, which makes it impossible to have rigorous control over factors that might affect anti-Müllerian hormone levels, since all cases were selected, including the ones with diseases that might alter AMH levels.

## CONCLUSIONS

Anti-Müllerian hormone levels correlated with embryo quality on Day 5, but had no correlation with embryo quality on Day 3 or pregnancy rate. The use of AMH to predict embryo quality still requires further studies, and AMH should be used only to assess ovarian reserve.
